# Effects of Surface Treatments and Cement Type on Shear Bond Strength between Titanium Alloy and All-Ceramic Materials

**DOI:** 10.3390/ma16186240

**Published:** 2023-09-15

**Authors:** Berkcan Tuncer, Guliz Aktas, Mustafa Baris Guncu, Diler Deniz, Mehmet Muhtarogullari, Nadin Al-Haj Husain, Mutlu Özcan

**Affiliations:** 1 Private Practitioner, Ankara 06100, Turkey; 2 Department of Prosthodontics, Faculty of Dentistry, Hacettepe University, Ankara 06100, Turkey; dtgulizaktas@gmail.com (G.A.); barisguncu@gmail.com (M.B.G.); cetin_diler@hotmail.com (D.D.); mmuhtar@hotmail.com (M.M.); 3 Clinic of Chewing Function Disturbances and Dental Biomaterials, Center of Dental Medicine, University of Zurich, 8032 Zurich, Switzerland; nalhaj88@gmail.com; 4 Department of Reconstructive Dentistry and Gerodontology, School of Dental Medicine, University of Bern, 3010 Bern, Switzerland

**Keywords:** aging, air abrasion, ceramic, dental materials, polymer-infiltrated ceramic network, prosthodontics, shear bond strength, titanium alloy

## Abstract

This study aimed to evaluate the effects of surface treatments and resin cement on the adhesion of ceramic and ceramic-like materials to titanium. A total of 40 specimens (5 mm diameter) of each material (lithium disilicate glass ceramic (LDGC—IPS e.maxCAD), lithium silicate glass ceramic (LSGC—VITA Suprinity) and a polymer-infiltrated ceramic network (PICN—Vita Enamic)) were fabricated using CAD/CAM technologies. In total, 120 titanium (Ti) specimens were divided into 12 groups, and half of the titanium specimens were tribochemically coated using CoJet. The titanium and all-ceramic specimens were cemented using either Self-curing adhesive cement (SCAC—Panavia 21) or a Self-curing luting composite (SCLC—Multilink Hybrid Abutment). After 5000 cycles of thermal aging, the shear bond strength (SBS) test was conducted using a universal testing machine. The failure modes of the specimens were analyzed using stereomicroscopy, and additionally, the representative specimens were observed using Scanning Electron Microscopy. ANOVA was used for the statistical analysis (*p* < 0.05). The post-hoc Duncan test was used to determine significant differences between the groups. The mean SBS values (mean ± STD) ranged from 15 ± 2 MPa to 29 ± 6 MPa. Significantly higher SBS values were acquired when the titanium surface was tribochemically coated (*p* < 0.05). The SCLC showed higher SBS values compared to the SCAC. While the LDGC showed the highest SBS values, the PICN presented the lowest. The tribochemical coating on the cementation surfaces of the titanium increased the SBS values. The specimens cemented with the SCLC showed higher SBS values than those with the SCAC. Additionally, the SCLC cement revealed a more significant increase in SBS values when used with the LDGC. The material used for restoration has a high impact on SBS than those of the cement and surface conditioning.

## 1. Introduction

With increasing patient demand for metal-free restorations, different all-ceramic and ceramic-like materials represent an option due to their perfect aesthetics, biocompatibility, and low thermal conductivity [1,2]. To date, various ceramic systems, materials, and techniques have been developed to produce CAD/CAM crowns. Monolithic all-ceramic materials are frequently preferred, as they show pleasing aesthetics, marginal integrity, and fracture strength and solve concerns regarding veneer ceramics [2,3,4]. Such materials may also be suitable for dental implant restorations and alternative core or zirconia-based ceramic veneer restorations [5].

Implant-supported restorations are expected to show aesthetically satisfactory results, as well as biological and functional success. Although conventional titanium abutments are long-lasting due to their physical properties, they mainly cause aesthetic problems in patients with thin gingival biotypes. For this reason, all-ceramic restorations are considered as an alternative in highly aesthetic areas [6,7]. However, the one-piece zirconia abutment has high stress in the region which connects the implant platform, and this leads to failures [8]. In recent years, some companies have started to produce two-piece (hybrid) titanium abutments that combine the aesthetic properties of all-ceramic abutments with the compatibility and mechanical properties of prefabricated titanium abutments [9,10]. In these restorations, the contact area of the abutment with the implant is prefabricated from titanium, and the remaining part of the restoration is produced from all-ceramic material using a CAD/CAM system. Then, these two interfaces are cemented to each other [11]. In this way, the possible risk of peri-implantitis caused by residual cement after intraoral cementing is eliminated. In addition, clinical success over time could be influenced by the adhesive connection between the two components. This is a weakness of these two-part systems. Various surface modifications were made to strengthen the connection in some studies, which proved that these modifications can influence the retention force between the components of two-piece abutment systems [11,12]. These modifications are tribochemical silica coating, Al_2_O_3_ sandblasting and laser applications [13]. 

**Figure 1:**
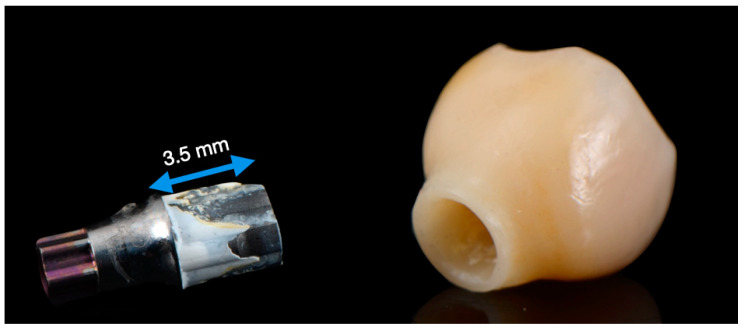
Debonded implant crown (**right**) and variobase (**left**). Traces of bonding cement are visible on the surface of the Variobase.

Resin cement is often used for the cementation of tooth structures due to its aesthetic and mechanical properties [14]. The polymerization mechanisms of resin cement are chemical, photo-polymerized, or dual. Chemical or dual-cure resin cement is generally recommended for two-piece titanium (hybrid) abutments, as various factors like cement chemical composition and the shade–thickness–opacity of ceramic materials affect cement polymerization in light-cured cement [15]. A luting agent is critical in two-piece abutment systems because of the strength and resistance of the ceramics. For this reason, cement selection should be carried out carefully (Figure 1) [2].

In dental literature, there are limited studies on the shear bond strength of various resin cements and tribochemical silica coating methods [9,11,13]. For this purpose, the current investigation aimed to evaluate the shear bond strength of resin cement types and tribochemical coating for grade V titanium abutments. Evaluations were carried out after thermal cycling using three different all-ceramic materials. The null hypothesis was formulated as follows: (1) the resin cements would show insignificant differences in shear bond strength depending on the material type; (2) the all-ceramic materials tested would not differ as a function of the type of resin cement, and (3) tribochemical coating would not affect the shear bond strength depending on the type of resin cement or ceramic material.

## 2. Materials and Methods

Grade V titanium specimens (N = 120) were fabricated using CAD/CAM procedures. The titanium alloy specimens were divided into three groups according to their ceramic materials (n = 40). The types of cement, ceramics and ceramic-like materials used in this study are listed in Table 1. 

**Figure 2:**
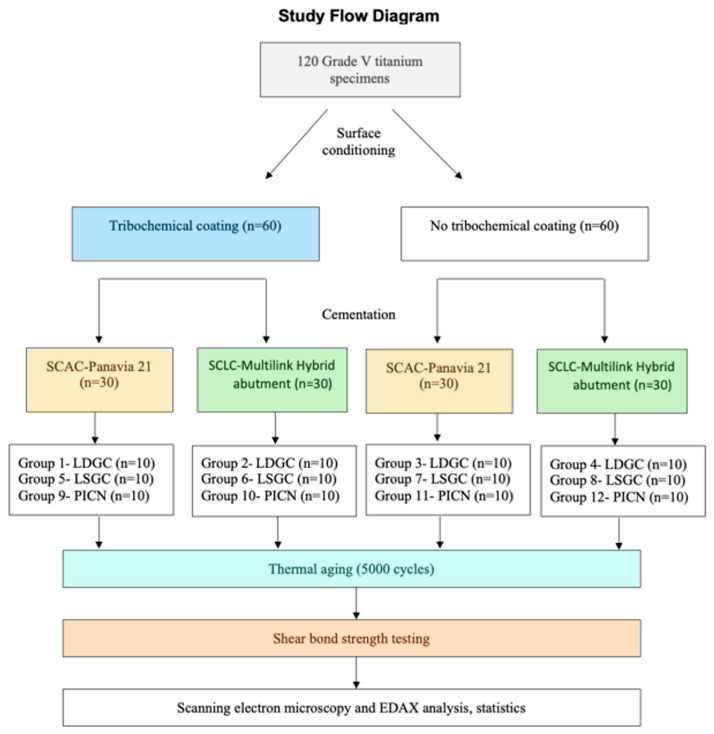
Illustration of the distribution of the experimental groups. SCLS: Self-Curing Luting Composite, SCAC: Self-Curing Adhesive Cement, LSGC: lithium silicate glass ceramic, LDGC: lithium disilicate glass ceramic, PICN: polymer-infiltrated ceramic network.

Half of each group were further divided into two sub-groups on the basis of the applied resin cements and surface treatments. A total of 12 different subgroups were produced (n = 10). The flow chart of the study is shown in Figure 2.

### 2.1. Preparation of Titanium Specimens

**Figure 3:**
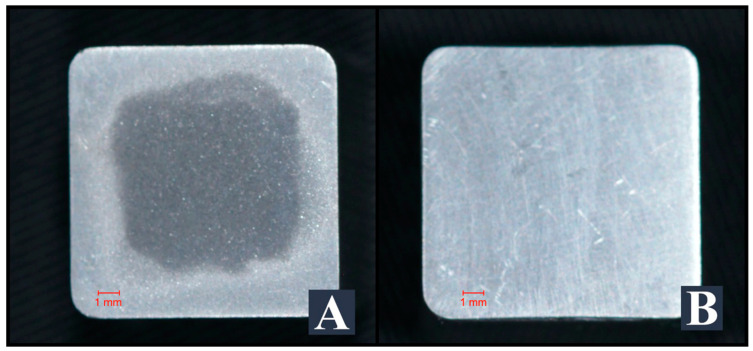
Tribochemically conditioned (**A**) and non-conditioned titanium specimens (**B**).

Titanium specimens (12 × 12 × 15 mm) were produced from grade V titanium. All surfaces of the specimens (12 mm × 12 mm) were manually polished to mimic the cementation surfaces of the implant abutments (Figure 1) using #600-, #800- and #1200-grit silicon carbide paper (Struers, Willich, Germany) under constant water cooling until a plain, shiny surface was obtained. Then, the specimens were cleaned in distilled water in an ultrasonic cleaning device for 5 min and dried with dry air. Based on the experimental groups in the study, tribochemical coating was applied to 60 titanium specimens with CoJet (3M ESPE, St. Paul, MN, USA) at a distance of 10 mm, at 2.8 bar pressure, for 15–20 s. No process other than polishing was applied to the other 60 titanium specimens (Figure 3).

### 2.2. Preparation of Ceramic Specimens

SGC, LDGC, and PICN specimens were obtained from CAD/CAM blocks (18 mm × 14.5 mm × 12.4 mm), and two cylinders with a diameter of 5 mm were designed perpendicular to the horizontal axis of each block using the Dental Wings 7 CAD program (Dental Wings Inc., Montreal, QC, Canada) and milled in Yenadent D30 CAM (Yenadent Ltd. Sti., İstanbul, Turkey). Each cylinder block was cut into 3 mm thick sections with a Metkon Microcut 201 (Metkon Industrial San. Tic. A.Ş., Bursa, Turkey). The surfaces of the specimens to be cemented to the titanium were polished with a Metkon Gripo 2V (Metkon Industrial San. Tic. A.Ş., Bursa, Turkey) polishing device.

The specimens were then cleaned in an ultrasonic cleaning device (Ultrasonic Cleaner Suc-110, Shofu, Kyoto, Japan) in distilled water for 5 min and air-dried. After applying the glaze liquid (IPS e.maxCAD Crystall/Glaze Liquid, Ivoclar Vivadent) to the surfaces not intended for cementing, the LDGC specimens were crystallized in a Programat P500 (Ivoclar Vivadent, Schaan, Liechtenstein) porcelain furnace according to the manufacturer’s recommendations. The LSGC specimens were crystallized in VITA v60 i-line (VITA Zahnfabrick, Bad Sackingen, Germany) porcelain, according to the manufacturer’s instructions, after glazing (VITA Akzent Plus glaze spray, VITA Zahnfabrick), similar to the LDGC specimens. No crystallization processes were carried out on the PICN specimens due to the resin in their structure. 

### 2.3. Cementation of Ceramic and Titanium Specimens

Before cementation, the ceramic specimens were cleaned in an ultrasonic cleaning device (Ultrasonic Cleaner Suc-110, Shofu, Kyoto, Japan) in distilled water for 5 min and air-dried. The LDGC and LSGC specimens were chemically etched with 5% hydrofluoric acid (IPS Ceramic Etching Gel, Ivoclar Vivadent, Schaan, Liechtenstein) for 20 s or, in the case of the PICN, for 60 s. A universal primer (Monobond Plus, Ivoclar Vivadent, Schaan, Liechtenstein) was applied to the cementation surface of each ceramic and titanium specimen until the surface had dried. The resin cement was prepared according to the manufacturer’s instructions and applied to the titanium and ceramic surfaces simultaneously. The ceramic specimens were placed on the titanium surface, finger pressure was applied for 60 s, and then the specimens were cured for 10 s with a LED light device (3M ESPE Elipar S10, St. Paul, MN, USA) at the center of the ceramic. Excess cement was carefully removed with a clean brush and probe, and Oxyguard II (Kuraray Noritake Dental Inc., Okayama, Japan) was applied around the specimens. After waiting for 7 min after cementation, the Oxyguard II was removed with air-water spraying, and the specimens were cured in a photo-polymerization device for 60 s (Solidilite V, Shofu Inc., Kyoto, Japan). 

### 2.4. Thermal Aging, Shear Bond Strength Testing and Scanning Electron Microscopy (SEM)

All specimens were placed in the thermocycler (SD Mechatronic GmbH, Westerham, Germany) in a metal wire basket for thermal aging for 5000 cycles at 5 ± 2 °C and 55 ± 2 °C. The temperature was chosen according to oral conditions and testing standards, simulating the oral environment. Subsequently, they were subjected to a shear bond strength test on a Lloyd LRX (Lloyd Instruments, Fareham, UK) universal testing machine at a 1 mm/min crosshead speed. After the shear bond strength test of the specimens was completed, one titanium and one ceramic from each group and two additional titanium specimens not used for cementation (one tribochemically coated and one control) were selected. Images of the specimens were captured using an FEI Quanta 400 FEG (FEI, Hillsboro, OR, USA) SEM device. The surfaces of the control titanium specimens were viewed at ×1000 magnification, and the surfaces of the titanium and ceramic specimens used in the study were viewed at ×250 magnification. Elemental analysis was performed on the images using the Energy-Dispersive Spectroscopy EDAX method.

### 2.5. Statistical Analysis

The data were statistically evaluated with IBM SPSS Statistics version 22 software. The Shapiro–Wilk test was used to examine whether the numerical data were normally distributed or not. In the case of 2 groups, a t-test was used for the independent groups, and in the case of 3 or more groups, one-way analysis of variance (one-way ANOVA) was used for the independent groups. The post-hoc Duncan test was used to determine the groups that showed differences in the one-way analysis of variance. The effects of roughening, cement type, and ceramic type on shear bond strength and the interactions of the variables were examined with a 2 × 2 × 3 factorial ANOVA, and *p* < 0.05 level was considered statistically significant.

## 3. Results

**Figure 4:**
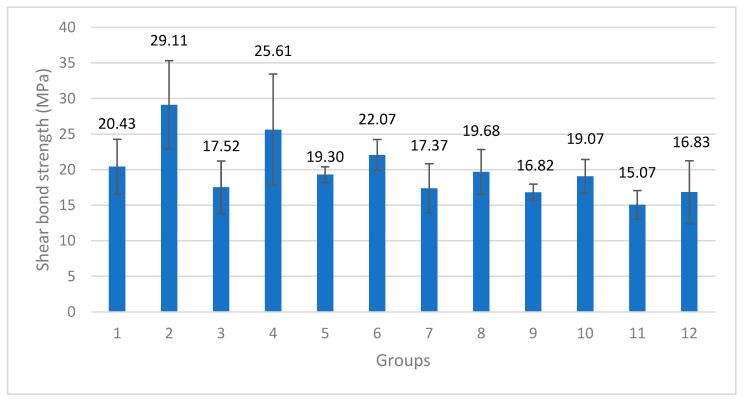
Mean shear bond Strength and standard deviation values of test groups according to one-way analysis of variance (MPa) (*p* = 0.001).

The bond strength values were normally distributed according to the Shapiro–Wilk test. According to the descriptive statistics of the one-way analysis of variance between independent groups, there was a statistically significant difference between the groups (*p* = 0.001) (Figure 4).

The mean shear bond strength value of 29.11 MPa in Group 2 was found to be statistically significantly higher than the values of all the other groups (*p* < 0.05), and the mean shear bond strength value of 25.61 MPa in Group 4 was significantly lower than that in Group 2 (*p* < 0.05) and higher than the values of the other groups (*p* < 0.05). The mean shear bond strength value of 22.07 MPa in Group 6 was found to be statistically significantly lower than the values of Group 2 and Group 4 (*p* < 0.05) but higher than those of Groups 3, 7, 12, 9 and 11 (*p* < 0.05). Groups 6, 1, 8, 5 and 10 performed similarly, while the shear bond strength values of Groups 1, 8, 5 and 10 were found to be statistically significantly lower than those of Group 2 and Group 4 (*p* < 0.05) and higher than the value of Group 11 (*p* < 0.05). The shear bond strength values of Groups 3, 7, 12 and 9 were statistically significantly lower than those of Groups 2, 4 and 6 (*p* < 0.05). A mean shear bond strength value of 15.07 MPa was obtained for Group 11. It was found to be statistically significantly lower than the values of Groups 2, 4, 6, 1, 8, 5 and 10 (*p* < 0.05). No statistically significant difference was found in Groups 3, 7, 12 and 9. A t-test was used to examine the shear bond strength values of the titanium samples with and without tribochemical coatings, regardless of the type of ceramic material or resin cement. The mean shear bond strength values of the specimens with tribochemical coatings on the titanium surface were found to be statistically significantly higher than the values of those without tribochemical coatings (*p* = 0.012). In the group with tribochemical coating, an average of 21.13 (±5.06) MPa was found, and in the group without tribochemical coating, a mean value of 18.68 (±5.48) MPa was found (*p* = 0.012). A t-test was used to examine the shear bond strength values only according to the resin cement used, regardless of whether tribochemical coating was applied and the type of ceramic used. For the shear bond strengths of the 60 specimens bonded with SCAC and SCLC, an average of 17.75 (±3.20) MPa was found in the specimens bonded with SCAC, with an average of 22.06 (±6.24) MPa for those with SCLC. In terms of shear bond strength for the two types of cement used, the mean shear bond strength values of the samples bonded with SCLC were found to be statistically significantly higher than the values of those bonded with SCAC (*p* = 0.001). 

**Figure 5:**
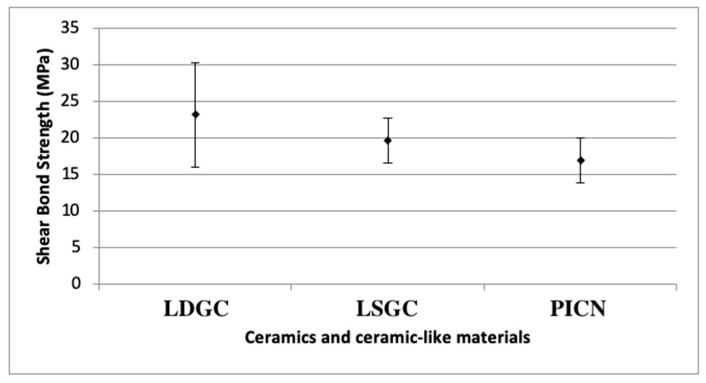
Shear bond strength results of ceramics and ceramic-like materials. LDGC: lithium disilicate glass ceramics, LSGC: lithium silicate glass ceramics, PICN: polymer-infiltrated ceramic network; SD: standard deviation.

One-way analysis of variance was used to examine the shear bond strength values only according to the ceramic materials, regardless of whether tribochemical coating was applied and the type of resin cement used. A statistically significant difference between the groups (*p* = 0.001) could be observed. The average shear bond strength value of 23.17 MPa obtained from the LDGC specimens was found to be statistically significantly higher than the values of the LSGC and PICN (*p* < 0.05). The average shear bond strength value of 19.61 MPa obtained for the LSGC specimens was found to be statistically significantly lower (*p* < 0.05) than the LDGC value and higher than the PICN value (*p* < 0.05) (Figure 5).

Tribochemical coating, cement type and ceramic material type affected shear bond strength significantly (*p* = 0.001). According to the partial eta square results, among these three factors, the ceramic material type (partial eta square values= 0.316 and 0.248) and cement type, respectively, have the greatest effects on shear bond strength. The effect of tribochemical coating (partial eta square value= 0.097) is lower than that of the ceramic material and cement type. The effect of cement type on shear bond strength showed a statistically significant difference when compared to the ceramic type (*p* = 0.001). According to the ANOVA analysis, it was observed that tribochemical coating increased the shear bond strength in a statistically significant manner (*p* = 0.001). It was found that cement type also affected shear bond strength in a statistically significantly way; when using the SCLC, a statistically significantly higher shear bond strength was obtained compared to that for SCAC (*p* = 0.001). It was found that the ceramic type had a statistically significant effect on the shear bond strength; the LDGC was found to have statistically significantly higher shear bond strength than the LSGC and PICN (*p* = 0.001). The LSGC was found to have a statistically significantly higher shear bond strength than the PICN (*p* = 0.001). These findings also support the t-test results.

The effect of the SCLC cement on shear bond strength resulted in statistically significant differences in effect between cement types and between ceramic types. While the mean shear bond strengths of the LDGC, LSGC and PICN specimens cemented with SCAC were 18.97 ± 3.96, 18.34 ± 2.70 and 15.94 ± 1.34 MPa, respectively, these values were 27.36 ± 7.11, 20.88 ± 2.91 and 17.95 ± 3.65 MPa when the SCLC was used. In the same order, the increases in shear bond strength were 8.39, 2.54 and 2.01 MPa. In the case of the LDGC, the effect of SCLC and SCAC was more significant than that of the LSGC and PICN (*p* = 0.001).

**Figure 6:**
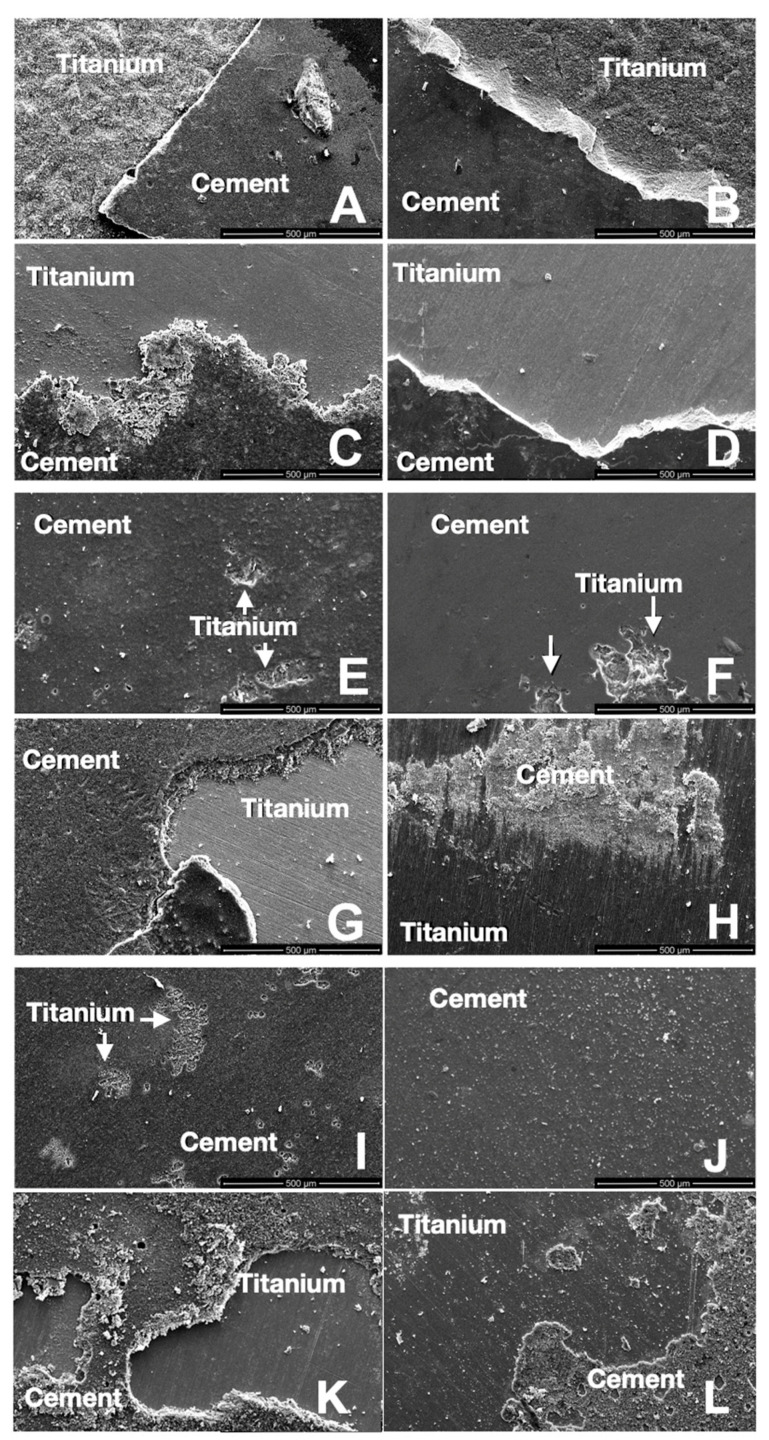
SEM images of titanium specimens after shear bond strength testing. LDGC groups 1–4 (Group 1 (**A**), Group 2 (**B**), Group 3 (**C**), Group 4 (**D**)); LSGC groups 5–8 (Group 5 (**E**), Group 6 (**F**), Group 7 (**G**), Group 8 (**H**)); and PICN groups 9–12 (Group 9 (**I**), Group 10 (**J**), Group 11 (**K**), Group 12 (**L**)). All even group numbers were treated with Panavia 21, and odd group numbers with Multilink Hybrid Abutment luting cement.

After the shear bond strength test was completed, one titanium and one ceramic specimen from each group were analyzed under a scanning electron microscope. A single researcher visually inspected all the other specimens. Mixed failure (cohesive and adhesive) was observed in the majority of the specimens (Figure 6).

**Figure 7:**
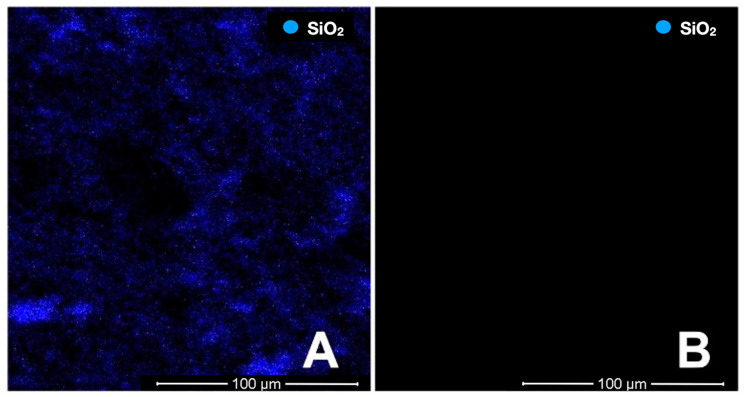
SiO_2_ distribution in the elemental examination of the non-cemented titanium samples with EDAX (the areas where the blue dots are concentrated show the places where SiO_2_ was detected). Tribochemical coating titanium sample (**A**), titanium specimen without tribochemical coating (**B**).

SEM imaging was performed on two titanium specimens, which were not used for cementation in order to observe the effect of the tribochemical coating on the surface. As a result of the elemental analysis conducted with the EDAX method on the SEM images captured at ×1000 magnification, SiO_2_ particles were also found on the tribochemically coated titanium surface (Figure 7).

## 4. Discussion

This study aimed to assess the effect of tribochemical coating on a grade V titanium abutment using two different types of cement and three different ceramics and ceramic-like materials. The null hypothesis was rejected because the tribochemical coating, ceramic material and cement type significantly affected the shear bond strength results.

The dental literature reported that one-piece zirconia abutments have mechanical disadvantages and that the marginal fit with the implant is not as good as that of titanium abutments [16]. As a result, many studies have shown the risk of undesirable long-term complications, such as the loosening of the abutment screw, wear at the implant–abutment interface and increasing bacterial colonization due to the gap in the implant–abutment contact area [17,18]. In a laboratory study published in 2017 by Rosentritt et al. [19], molar-shaped full anatomical crowns were fabricated from four different CAD/CAM materials on prefabricated titanium abutments. These crowns were manufactured with and without occlusal screw holes. After thermal cycling, a fracture test was applied to the specimens, and a mechanical fatigue test equivalent to 5 years of use was applied. The researchers reported that the crowns designed with occlusal screw holes in all four materials showed a lower fracture strength. This may be due to microcracks in the material during screw hole milling and supports the production of restorations from blocks with self-occlusal screw holes. For these reasons, this study evaluated the performance of two-piece titanium hybrid abutments and ceramic and ceramic-like materials [20,21,22].

For dental implants, both grade IV and grade V titanium can be used according to the clinical demands. While grade IV consists of unalloyed titanium with trace amounts of carbon, nitrogen, oxygen and iron, grade V is composed of 90% titanium, 6% aluminum and 4% vanadium. In clinical cases where enhanced mechanical properties are required, grade V titanium is preferred and was therefore used in this study [23].

The most important factor in the two-piece titanium abutment is the stability of the connection between the restoration material and the abutment. Several factors affect the connection, like the resin cement, restoration material or abutment surface treatment. Fonseca et al. [24], in a study published in 2012, investigated the effects of sandblasting and additional surface treatments applied to a titanium surface. After sandblasting with different particle sizes of Al_2_O_3_ (50, 120 and 250 μm) or tribochemically coating the titanium discs, additional surface treatments were applied to each group separately. It was reported that the highest bond strength values were obtained after air-abrasion with 250 μm particle size Al_2_O_3_ and in the groups with tribochemical coating. In another study, the authors investigated the influence of different airborne particle abrasion (APA) methods for the Ti-base surface on the stability of the bonded interface. According to that study, 50 μm Al_2_O_3_ provided the most stable bonded interface among the different treatments [25]. Özcan and et al. investigated whether the bonding of the composite to the titanium surface was affected by the application of different roughening methods to the titanium surface and the washing of the surface with water after the applied roughening processes [26]. Similar to the aforementioned studies, this study found that tribochemical coating of the titanium surface increased the shear bond strength.

Another important factor in the connection between restoration and abutment is the type of cement. In 2021, Dhesi et al. [10] investigated the effects of five different ceramic and hybrid materials and three different cements and thermal cycles. According to the results, regardless of the restoration material, differences in the shear bond strength values of the cements were found in the Multilink Hybrid Abutment and Panavia 21 cement, respectively. In another study conducted in 2018, the adhesion of lithium disilicate ceramics to titanium was investigated using various surface treatments and cements. The self-cured luting composite showed better shear bond stress than the self-cured adhesive cement in that study [27]. In our research, two types of cement were used to bond the titanium disks to the three different ceramic materials. The selection of the SCAC (Panavia 21) was due to its ability to provide a higher bond strength compared to the other cements, and the SCLC (Multilink Hybrid Abutment) was chosen as a cement reserved for extraoral use alone and for bonding titanium to ceramics in hybrid abutments. Titanium and ceramic specimens were cemented in these cements and subjected to the thermal cycle, and then the shear bond stress was evaluated. In the study of Dhesi et al., similar to this study, the shear bond strength values of the cements were found to be higher in the case of SCLC than in SCAC. Also, in another study in 2022, the Multilink Hybrid Abutment cement was shown to have better biological properties and lower cytotoxicity. hence, with respect to all of these properties, the Multilink Hybrid Abutment cement was preferred for cementing implant crowns on abutments [28]. Differences in composition, especially the quantities of photo-initiators and chemicals, monomeric composition, filler content and the ratio of diluent monomers, can cause the observed changes in performance.

Recently, different types of ceramic materials, such as lithium silicate glass ceramic, lithium disilicate glass ceramic and polymer-infiltrated ceramic networks, were employed for chairside fabrication and preventing different failures. However, each material has its advantages and disadvantages [29]. In an in vitro study, four different CAD/CAM materials (IPS e.maxCAD, Celtra Duo, Lava Ultimate, VITA Enamic) were investigated in terms of their bond strengths with resin cement through microtensile testing after applying different surface treatments. The researchers found that the bond strength values were higher for the lithium silicate glass ceramic (Celtra Duo) and lithium disilicate glass ceramic (IPS e.maxCAD) than the polymer-infiltrated ceramic network (VITA Enamic) and resin nano ceramic (Lava Ultimate) [30]. Similar to that study, lower shear bond strength values were observed for the polymer-infiltrated ceramic network (VITA Enamic) compared to the lithium silicate glass ceramic (Vita Suprinity) and lithium disilicate glass ceramic (IPS e.maxCAD) in the current study.

According to the dental literature, various types of failure were encountered when the cement interfaces of ceramic and ceramic-like materials were examined after shear bond strength testing in two-piece abutments. In another investigation on the adhesion of five different ceramics and ceramic-like materials with three different resin types of cement, adhesive and cohesive failures after shear bond strength testing were observed at similar frequencies [10]. In another study examining the bond strength of lithium disilicate ceramics and zirconium ceramics with two-piece abutments, mixed failure (adhesive and cohesive) was observed in the lithium disilicate samples [31]. In this study, mixed failure was found in all the specimens, similar to the literature.

To obtain results quicker and more easily, ensuring easier control and standardization of the test conditions, laboratory studies are frequently used to test the current materials and treatment methods. However, in vivo validation, sintering protocols [32], patient-related factors, dynamic loads and fatigue due to the material used are limitations of this study, and its findings should be supported through clinical studies. Further limitations include the use of type V titanium alone, one type of thermal cycling and a universal testing machine.

## 5. Conclusions

According to the results of this study, it can be concluded that regardless of the cement or restoration material, applying tribochemical coating to the cementation surface of grade V titanium significantly increases the shear bond strength. Furthermore, the choice of ceramic material and cement type has a significant impact, as the self-cured Multilink Hybrid Abutment showed better results than the self-cured adhesive cement Panavia 21. Ranking from higher to lower, we determined the order as lithium disilicate ceramic, lithium silicate ceramic and the resin-infiltrated ceramic network, with mean values ranging from 22 to 42 MPa. The evaluation of the failure patterns emphasizes the importance of a strong and stable connection between the abutment and restoration to prevent failures compromising longevity. 

## Figures and Tables

**Table 1:** Description of cements, ceramics and ceramic-like materials used in this study (according to the manufacturers).

Classification	Brand	Composition	Code	Manufacturer
Self-curing Luting Composite	Multilink Hybrid Abutment	Dimethacrylate, HEMA, barium glass, ytterbium trifluoride, spheroid mixed oxide, titanium oxide	SCLC	Ivoclar Vivadent, Schaan, Liechtenstein
Self-curing Adhesive Cement	Panavia 21	Hydrophobic aromatic dimethacrylate, hydrophilic alipathic dimethaccrylate, MDP, fillers, BPO, hydrophilic dimethacrylate, DEPT, sodium aromatic sulfonate	SCAC	Kuraray Noritake Dental Inc., Okayama, Japan
Lithium silicate glass ceramic	Vita Suprinity	8–12% zirconia, 56–64% silicon dioxide, 15–21% lithium oxide, 0.1 lanthanum oxide, <10 pigments, >various	LSGC	VITA Zahnfabrik, H. Rauter GmbH & Co. KG, Bad Säckingen, Germany
Lithium disilicate glass ceramic	IPS e.max CAD	58–80% silicon dioxide, 11–19% lithium oxide, 0–13% potassium oxide, 0–8% zirconium dioxide, 0–5% aluminum oxide	LDGC	Ivoclar Vivadent, Schaan, Liechtenstein
Polymer-infiltrated ceramic network	Vita Enamic	86% *w*/*w* fine-structure feldspathic ceramic (58–63% silicon dioxide, 20–23% aluminum oxide, 9–11% sodium dioxide, 4–6% potassium oxide, and 0–1% zirconium dioxide) 14% polymer (UDMA and TEGDMA)	PICN	VITA Zahnfabrik, H. Rauter GmbH & Co. KG, Bad Säckingen, Germany

## Data Availability

Not applicable.
